# From ECG to Imaging: Challenges in the Diagnosis of Adult Congenital Heart Diseases

**DOI:** 10.3390/jcm13164865

**Published:** 2024-08-18

**Authors:** Simina Crișan, Ruxandra-Maria Băghină, Silvia Luca, Oana Pătru, Mihai-Andrei Lazăr, Cristina Văcărescu, Marius Rus, Dragoș Cozma, Dan Gaiță, Constantin-Tudor Luca

**Affiliations:** 1 Cardiology Department, “Victor Babes” University of Medicine and Pharmacy, 2 Eftimie Murgu Sq., 300041 Timisoara, Romania; simina.crisan@umft.ro (S.C.); ruxandra.croicu@umft.ro (R.-M.B.); silvia.luca0@student.umft.ro (S.L.); oana.patru@umft.ro (O.P.); lazar.mihai@umft.ro (M.-A.L.); dragos.cozma@umft.ro (D.C.); dgaita@cardiologie.ro (D.G.); constantin.luca@umft.ro (C.-T.L.); 2 Institute of Cardiovascular Diseases Timisoara, 13A Gheorghe Adam Street, 300310 Timisoara, Romania; 3 Research Center of the Institute of Cardiovascular Diseases Timisoara, 13A Gheorghe Adam Street, 300310 Timisoara, Romania; 4 Department of Medical Disciplines, Faculty of Medicine and Pharmacy, University of Oradea, 410073 Oradea, Romania; rusmarius@uoradea.ro

**Keywords:** congenital heart disease, Ebstein’s anomaly, atrial septal defect, ECG, imaging

## Abstract

Congenital heart diseases (CHD) are one of the most common birth defects and the main leading cause of death in children. Many patients with CHD are reaching adulthood due to the success of improved contemporary surgical procedures. Understanding the etiology of CHD remains important for patient clinical management. Both genetic and environmental factors are involved in the development and progression of CHD. Variations in many different genes and chromosomal anomalies can be associated with CHD, by expression of different mechanisms. Sporadic cases are the most frequently encountered in these patients. Atrial septal defect is a common congenital heart disease that refers to direct communication between atrial chambers, found isolated or associated with other syndromes. Imaging techniques, especially transthoracic and transesophageal echocardiography (TOE) represent the key for diagnosis and management of ASD. The disease has a major incidence in adulthood, due to late symptomatology, but assessment and treatment are important to avoid time-related complications. Ebstein’s anomaly is a rare congenital disease, with a dominant genetic participation, characterized by an abnormal displacement of the tricuspid valve and right ventricular myopathy, often requiring surgical intervention. Alongside echocardiography, cardiac magnetic resonance (CMR) imaging is the gold standard tool for the assessment of ventricular volumes. Early diagnosis and adequate treatment are mandatory to avoid possible complications of CHD, and thus, ECG, as well as imaging techniques, are important diagnostic tools. However, patients with CHD need a special healthcare team for the entire monitorization in various life stages.

## 1. Introduction

Congenital heart diseases (CHD) are one of the most common birth defects and occur in 1% of liveborn children [1]. Although nowadays, up to 90% of children with CHD survive and reach maturity, the pathology remains the leading cause of birth-defect-related death in childhood [2]. The etiology is multifactorial, including genetic and environmental contributors. However, in 60% of cases of CHD, the etiology is unknown. Many studies estimated that more than 400 genes are linked to CHD. The main genetic changes associated with CHD are mutations in genes encoding transcription factors and cell signaling transducers that interfere with cell-type specification and differentiation, causing alterations related to the structure and function of the heart [3]. The genetic causes of CHD include chromosomal anomalies, aneuploidies, and copy number variants [4]. Genetic etiology divides CHD into non-syndromic CHD and syndromic CHD. Non-Syndromic CHD encompasses isolated congenital abnormalities of the heart. Atrial septal defect (ASD) is the most common congenital disease diagnosed in adulthood, found in both syndromic and non-syndromic CHD. Due to anatomy heterogeneity and time-related complications, many challenges in optimal diagnosis and treatment remain to be overcome [5]. Genetic involvement has an important role in the early morphogenesis of cardiac structures. In Ebstein’s anomaly, duplication of the 15q chromosome affects the formation of the tricuspid valve, leading to abnormal apically displaced leaflets. The diagnosis of CHD can be challenging, requiring physical examination and multiple paraclinical investigations. Patients with CHD may develop cardiac complications such as arrhythmias, heart failure, and valve insufficiency, even after surgical correction of the structural abnormalities. Most congenital heart defects are not curable and require lifelong specialized care [6]. Since atrial septal defect (ASD) and Ebstein’s anomaly are the most common CHD that we have found in our clinical practice, we discuss data from the current literature, as well as two brief case presentations, regarding this pathology.

## 2. Atrial Septal Defect (ASD)

Atrial septal defect (ASD) is the most common congenital abnormality, defined as a persistent interatrial communication, which allows blood shunting between the systemic and the pulmonary circulation [7]. ASD is found in 13% of cases of congenital heart diseases with a high prevalence in female patients. ASD is frequently detected in adulthood due to its late symptomatology and pathophysiological consequences [8].

The clinical features are related to the anatomic location, type, and size of ASD. Ostium secundum atrial septal defect (OSASD) is the most common type, found in approximately 80% of ASD cases, with interatrial communication located in the region of the fossa ovalis [9]. Ostium primum (OP), or atrioventricular atrial septal defect (AVASD), is defined as a communication near the origin of the tricuspid and mitral valve, creating an atrioventricular canal. It is often associated with abnormalities of atrioventricular valves and Down Syndrome [10]. Superior sinus venosus atrial septal defect (SVASD) and inferior sinus venosus atrial septal defect (IVASD) are located near the entry of the superior vena cava (SVC) or inferior vena cava (IVC). SVASD represents 5% of all types of ASD and is often associated with partial anomalous pulmonary venous drainage from the right lung [11]. Unroofed coronary sinus is the rarest ASD, found in less than 1% of cases, representing a communication between the left and the right atrium through the ostium of the coronary sinus. Depending on the extent of unroofing of the coronary sinus, the association with persistent left superior vena cava (PLSVC) can lead to mild systemic desaturation [12]. A more particular situation is represented by multiple defects of the interatrial septum, a situation that may be found in up to 10% of all OSASD. Beyond a great variant of morphological types, from two defects present within the interatrial septum, associated with right ventricular overload, to multiple small defects or fenestration, with or without evidence of right ventricular overload, the main issue is that in terms of therapy, these multiple defects are more difficult to close with the transcatheter approach. In this case, percutaneous closure may be performed using a single centrally deployed septal or cribriform occluder or by using multiple devices [13].

The symptomatology of patients with ASD depends on the size of the defect. Patients with very small ASD are asymptomatic, while patients with larger defects, up to 10 mm, develop symptoms in the third decade [14].The most common clinical presentation of patients with ASD includes dyspnea, exercise intolerance, fatigue, or palpitations due to volume overload of the right ventricle (RV) and pulmonary hypertension (PH) [8,13]. In rare cases, the first manifestation can be a transient ischemic attack, or acute limb ischemia, due to paradoxical and peripheral arterial embolization. The prevalence of patients with ASD who associate paradoxical embolism is up to 14%. The risk factors for paradoxical embolism are deep venous thrombosis and atrial fibrillation. In ASD patients, pulmonary hypertension is associated with poor prognosis and a high risk of atrial arrhythmia and right heart failure development. A physical examination can reveal systolic flow murmur in the pulmonary valve region due to increased pulmonary flow, splitting of the second heart sound, and signs of right ventricle failure due to overload volume of the RV, such as hepatomegaly, elevated jugular venous distention, or peripheral edema. If Eisenmenger’s syndrome develops, cyanosis and clubbing are present [15].

Electrocardiogram (ECG) findings are related to right heart failure and right atrial and ventricular enlargement. Signs of a hemodynamically significant left-to-right shunt may include right axis deviation, first-degree heart block, right bundle branch block, or right ventricular hypertrophy. The most common arrhythmias that may complicate ADS are atrial fibrillation, atrial flutter, and junctional rhythms. In patients with hemodynamically significant shunt, the rSR’ pattern in precordial leads V1 or V2 revealed on ECG is correlated to the RV enlargement and the size of the defect. Severe right axis deviation, right ventricular hypertrophy, and extensive repolarization abnormalities may be seen in the presence of an ASD with Eisenmenger’s syndrome [7].

Transthoracic echocardiography (TTE) is the key for diagnosis and management of ASD in order to identify the size and type of defect, shunt direction, as well as an accurate evaluation of the right heart. As a first-line diagnosis tool, TTE can provide comprehensive information of the hemodynamic consequences of the defect. For suspected shunt lesions, TTE uses the ratio of the pulmonary to systemic shunt volume (Qp/Qs) as an established method. A shunt ratio above 1.5 is considered hemodynamically significant. For accurate diagnosis, transesophageal echocardiography (TOE) provides precise visualization of the interatrial septum, including the residual septum’s morphology and the rim’s size and quality. Additional imaging, such as cardiac magnetic resonance (CMR) or computed tomography (CT), are able to exclude associated cardiac anomalies such as anomalous pulmonary venous drainage or unexpected defects not seen on TTE or TOE [16].

The first-line treatment for OSASD is device closure. The minimum criteria for this type of treatment include the presence of a diameter of the defect ≤ 38 mm and a sufficient rim of 5 mm (atrial septal tissue around the defect), excepting the rim from the aorta [9]. The recent studies confirmed that transcatheter atrial septal defect closure has a lower risk of mortality, perioperative stroke, and post-procedural atrial fibrillation compared to traditional surgery [17]. The meta-analysis data have proven that the Amplatzer occluder device is superior to the CardioSEAL/STARFlex occluder, regarding a better prognosis and fewer complications [18]. For patients with PH, the calculation of pulmonary vascular resistance (PVR) is necessary. Device closure in patients with PVR < 5 WU can improve the symptomatology and decrease pulmonary arterial pressure (PAP). For patients with Eisenmenger’s physiology or with pulmonary arterial hypertension and PVR ≥ 5 WU, the ASD closure is not recommended. Surgical intervention has low mortality and a good prognosis for young patients with no PH. However, for advanced age patients, individual surgical risk due to comorbidities must be carefully weighed against the potential benefits of ASD closure [9].

### Atrial Septal Defect—Case Presentation

**Figure 1:**
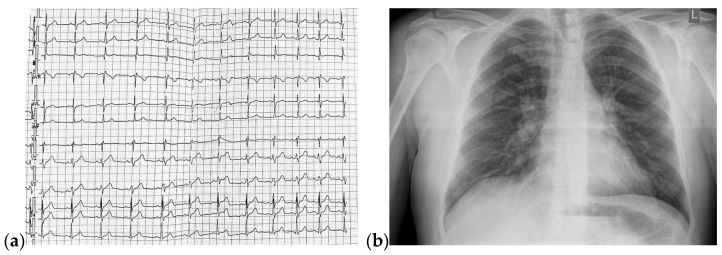
(**a**) 12-lead ECG revealed sinus rhythm and rSR’ pattern in precordial leads V1 or V2; (**b**) Postero-anterior chest view radiography showed hilar bilateral enlargement and increased vascular density in the upper half of the hilum. Abbreviations: ECG—electrocardiogram.

A 36-year-old non-smoker male patient, without cardiological follow-up and no cardio-active medication at home, presented with dyspnea, mild effort intolerance, and headache. Physical examination revealed peripheral cyanosis, body mass index (BMI) = 27.68 kg/m^2^, blood pressure (BP) = 115/70 mmHg, heart rate (HR) = 75 bpm, and unremarkable cardiac and pulmonary auscultation. The electrocardiogram showed sinus rhythm, HR = 75 bpm, rSR’ pattern in precordial leads V1 or V2 (Figure 1a). The chest radiography revealed a normal heart size with hilar bilateral enlargement and increased vascular density in the upper half of the hilum (Figure 1b).

**Figure 2:**
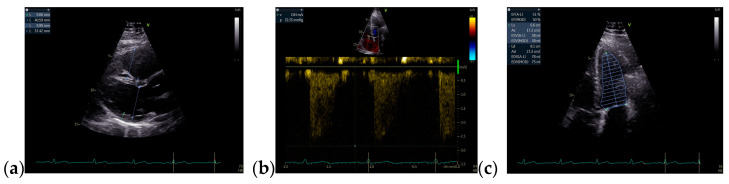
TTE on admission: (**a**) Parasternal long axis view with enlarged diameter of RV; (**b**) Apical 4-chamber view, tricuspid continuous wave Doppler flow, with a maximum RV-RA gradient of 32.33 mmHg; (**c**) Apical 4-chamber view, EF = 51% (Simpson’s method); Abbreviations: TTE—transthoracic echocardiography; RV—right ventricle; RA—right atrium; EF—ejection fraction.

**Figure 3:**
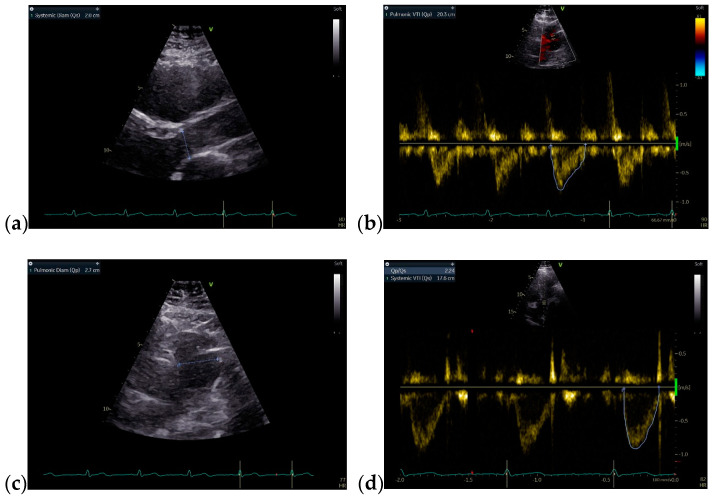
TTE on admission: (**a**) Parasternal long axis view measuring LVOT diameter = 2 cm; (**b**) Parasternal short axis view: Pulmonic VTI = 20.3 cm; (**c**) Parasternal short axis view: pulmonary artery trunk = 2.2 cm; (**d**) Apical 5 chamber view: Systemic VTI = 17.6 cm, Qp/Qs = 2.24; Abbreviations: TTE—transthoracic echocardiography; LVOT—left ventricle outflow tract; VTI—velocity-time integral.

The TTE performed on admission showed a non-dilated left ventricle with an ejection fraction (EF) of 51% (using Simpson’s method), dilated right chambers, mild functional tricuspid regurgitation, mild pulmonary hypertension, tricuspid annular plane systolic excursion of 19 mm, inferior vena cava (IVC) with 18 mm diameter, and respiratory variability greater than 50%. The TTE raised the suspicion of ASD with significant hemodynamic shunt (Qp/Qs = 2.24) (Figure 2 and Figure 3).

**Figure 4:**
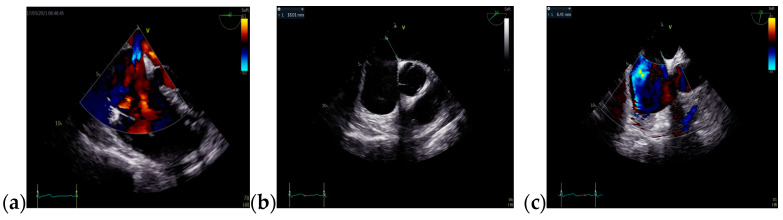
TOE: (**a**) Mid-esophageal 4-chamber view: turbulent color Doppler flow at the level of the interatrial septum; (**b**) Mid-esophageal short-axis view: atrial septal defect with a diameter of 1.8 cm (18 mm); (**c**) Mid-esophageal short-axis view: aortic rim = 6.4 mm. Abbreviations: TOE—Transesophageal echocardiography.

Due to the patient’s symptomatology, clinical features. and suspected findings from the TTE, including increased Qp/Qs ratio, a TOE was required and revealed an OSASD, with a diameter of 1.8 cm and aortic rim of 6.4 mm (Figure 4).

**Figure 5:**
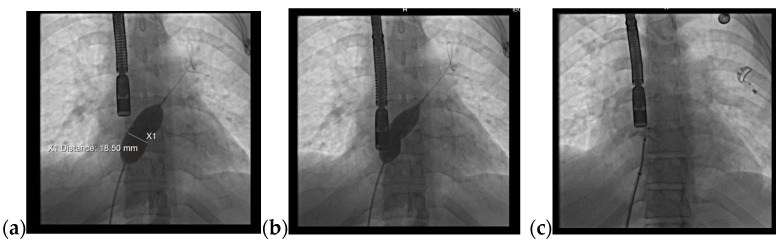
Transcatheter device closure: (**a**,**b**) Fluoroscopy, measurement of the defect with a diameter of 18.5 mm; (**c**) implant of a 20 mm Amplatzer device.

Considering the hemodynamic challenges of the RV and increased Qp/Qs ratio, the recommended treatment of the defect was based on transcatheter device closure (Figure 5a). In order to establish the perfect diameter of the closing device, the diameter of the defect can be measured by ultrasound, when 25% of the determined maximum value is added, or by fluoroscopy, when 2 mm are added. Subsequently, the ASD was percutaneously closed with an ASD^®^ Amplatzer septal occluder prostheses 9-ASD-020 without any residual shunt or complications (Figure 5b,c).

**Figure 6:**
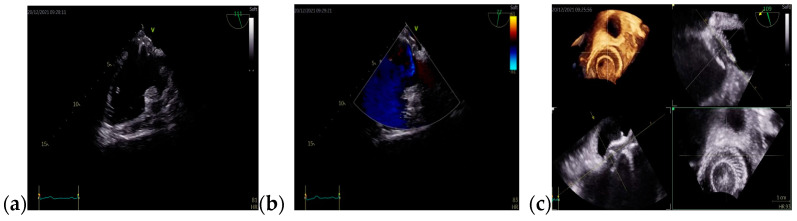
TOE: (**a**) Bicaval view: the Amplatzer occluder device (**b**) Mid-esophageal short-axis view: Amplatzer occluder device with no residual shunt (**c**) 3D TOE: Amplatzer occluder device; Abbreviations: TOE—transesophageal echocardiography.

The patient’s symptomatology improved consistently in the immediate postoperative period. Four months later, the TOE revealed the Amplatzer occluder device located at the level of the interatrial septum, with no residual shunt (Figure 6).

## 3. Ebstein’s Anomaly

Ebstein’s anomaly is a rare valvular disorder found in 1% of cases of congenital heart diseases, defined by the abnormal apical displacement of the tricuspid valve, tethering of the septal and posterior leaflets to the myocardium, and atrialization of the inlet portion of the right ventricle [19]. The anomaly is more common in patients with a family history of CHD, especially in twins and in those with maternal exposure to benzodiazepines. The genetic implication due to the duplication of the 15q chromosome affects the early morphogenesis of the tricuspid valve, causing a failure of delamination of the tricuspid valve leaflets from the interventricular septum. In normal conditions, the atrioventricular valves are formed by a process of delamination of the inner layers of the inlet zone of the ventricles, and the leaflets develop equally from the endocardial cushion tissues and the myocardium [20,21]. In Ebstein’s anomaly, it is assumed that the delamination of the tricuspid valve leaflets fails to occur, but the exact mechanism is not entirely understood [22]. Due to the apical displacement of the tricuspid valve, the RV is divided into an atrialized and a functional part. Due to fibrosis and myopathy, the atrialized RV is often dyskinetic. Depending on its severity, the systolic regurgitation from the functional RV through the tricuspid valve may lead to hemodynamic changes due to dilation of the RA and atrialized RV [23,24,25].

Cases in 80% of patients with Ebstein’s anomaly may be associated with ASD or a patent foramen ovale. In these situations, paradoxical emboli can occur due to patent shunts. In approximately 10% of cases, the anomaly is associated with ventricular septal defects and pulmonary atresia. These associated pathologies can worsen the prognosis of patients with Ebstein’s anomaly [26]. In approximately 15% to 50% of cases of patients with congenitally corrected transposition of the great arteries, the abnormal tricuspid valve fulfills the criteria for Ebstein’s anomaly [27].

The clinical presentation depends on the hemodynamic abnormalities. In mild forms of the disease, patients can be asymptomatic for a long period. Most of the clinical presentations can include cyanosis and right-sided heart failure symptoms. In rare cases, arrhythmias and sudden cardiac death can be the first manifestations of the disease. The clinical examination may reveal dyspnea, poor exercise tolerance, or chest pain. Although symptomatic children with Ebstein’s anomaly may have progressive right-sided heart failure, most of them reach adolescence and adulthood [28].

The prenatal diagnosis of Ebstein’s anomaly is rarely reported in mild or rare pregnancies since anomalies of the tricuspid valve may be detected in fetuses as early as the 14th week of gestation. However, despite a normal heart examination in early or middle pregnancy, the anomaly may only be detected in the newborn [29]. The prognosis and survival rate for neonates with Ebstein’s anomaly continues to improve due to conservative therapy and surgical strategies. Fetal distress and pulmonary atresia or stenosis are the main risk factors for mortality among newborns with Ebstein’s anomaly, while factors associated with death in childhood and adolescence are represented by a young age at diagnosis (<12 months), hepatomegaly, mechanical ventilation, and pulmonary valve defects. Compared to Ebstein’s anomaly diagnosed at an early age, a relatively better prognosis is expected for the adult population diagnosed with this condition. In this situation, the clinical outcome may be influenced by functional and anatomical factors like cyanosis, NYHA functional class at the time of diagnosis, ECG features, and the presence of atrial tachyarrhythmia [30].

The ECG of a patient with Ebstein’s anomaly can reveal multiple abnormal findings. The most common ECG pattern includes right atrial enlargement, with “Himalayan P wave”; right bundle branch block, often with notched QRS complexes as a result of an infra-Hisian conduction disturbance and abnormal activation of the atrialized RV; and PR prolongation due to atrial dilation and delayed intra-atrial conduction [31]. In many cases, the ECG can reveal a first-degree AV block as a consequence of RA enlargement with long interatrial conduction time [32]. A third-degree AV block is considered a rare complication of Ebstein’s anomaly. In addition, wide QRS tachycardia over a septal accessory atrioventricular pathway, ventricular tachycardia, atrial flutter, and atrial fibrillation can occur [33].

TTE is the most useful, non-invasive method for the diagnosis of Ebstein’s anomaly, providing an accurate evaluation of the tricuspid valve leaflets and evaluating the size and function of cardiac chambers [34]. TTE can reveal the apical distal displacement and tethering of the septal or posterior leaflet, as well as the dimensions of the atrialized and functional remaining RV. A displacement index of >8 mm/m^2^ supports the diagnosis of Ebstein’s anomaly and may be used to differentiate the condition from other right heart disorders [35]. Both anterior and septal leaflets are generally assessed from the apical 4-chamber view. The inferior (or posterior) leaflet is best seen from an RV inflow view, a parasternal long-axis view with angulation, or from the subcostal long-axis view. A color Doppler flow is used to confirm the tricuspid regurgitation [36,37].

TOE is used for intra-operative assessment and, rarely, in patients with limited acoustic windows. TOE provides information regarding the tricuspid valve from RA and RV perspectives, allowing a better understanding of leaflet morphology and mobility. Mid-esophageal 4-chamber and trans-gastric sagittal views can demonstrate the apical displacement of the functional tricuspid valve annulus. Furthermore, the 4-chamber view can assess the anterior and septal leaflet attachments to the myocardium. A mid-esophageal oblique view can reveal the anterior rotation of the functional tricuspid valve orifice into the RV outflow tract. ASD and patent foramen ovale can also be assessed in this view, as well as from the bicaval or 4-chamber view [32].

Cardiac magnetic resonance (CMR) imaging remains the gold standard tool for the assessment of ventricular volumes. In Ebstein’s anomaly, CMR can be used in order to achieve the ventricular size and function when echocardiographic image quality is inadequate. CMR achieves a stack of steady-state free precession cardiac images in planes axial to the body or in short-axis to the heart [38]. The regurgitant volume of the tricuspid valve is calculated by removing the volume measured by velocity mapping of the pulmonary artery from the total RV volume measured on the cine images [39]. Before and after surgery, CMR represents a valuable prognostic tool for the before and after surgery evaluation, revealing unrestricted views for the assessment and quantification of the dilated right heart and RV function [40].

The therapeutic management of patients with Ebstein’s anomaly relays on symptomatology. In many cases, the treatment is supportive in order to reduce pulmonary vascular resistance and hypoxemia. Right heart failure symptoms are treated with loop diuretics and guideline medical therapy. Anticoagulation therapy is recommended for patients with paradoxical emboli or atrial fibrillation. Symptomatic rhythm disorders can be treated conservatively by using antiarrhythmic medication. However, most of these patients usually need definitive ablative therapy for supraventricular arrhythmias. A cardiac pacemaker implant can be used for patients who develop AV block or bradyarrhythmia [41].

Surgical treatment repair remains challenging and should only be performed by surgeons with experience. The preferred surgical therapy is represented by tricuspid valve repair along with the closure of the potential atrial septal defect. The Glenn procedure is recommended for patients with severe RV dilation and moderate RV dysfunction. Bidirectional cavo-pulmonary anastomosis is required in order to provide RV volume or pressure offloading. The main contraindications for the procedure include mean PAP > 20 mm Hg, PVR > 4 WU, and elevated left ventricular end-diastolic pressure or left atrial pressure [42,43]. The da Silva Cone procedure is the surgical approach preferred in young children and adults and consists of mobilizing the anterior and posterior leaflets from their anomalous attachments, rotating the detached edges of leaflets, and suturing them to the septal edge of the anterior leaflet at the level of the tricuspid valve annulus [44]. The Danielson repair consists of repairing the tricuspid valve by plication of the atrialized part of the RV, narrowing the size of the tricuspid valve and creating a mono-leaflet competent tricuspid valve. The Carpentier repair consists of plication of the atrialized portion of RV and narrowing the tricuspid valve annulus, but in the direction of right angles from that performed by Danielson [43,45]. In specialized centers, the post-operative mortality is currently 6%. Approximately 97% of patients with Ebstein’s anomaly have a survival rate over 5 to 10 years following surgical procedures [46,47]. However, heart failure has an important contribution to late morbidity and mortality in adult CHD. Despite these incredible improvements in surgical repair options, in some cases, due to severe complications, heart transplantation may be the only option [48,49].

### Ebstein’s Anomaly—Case Presentation

**Figure 7:**
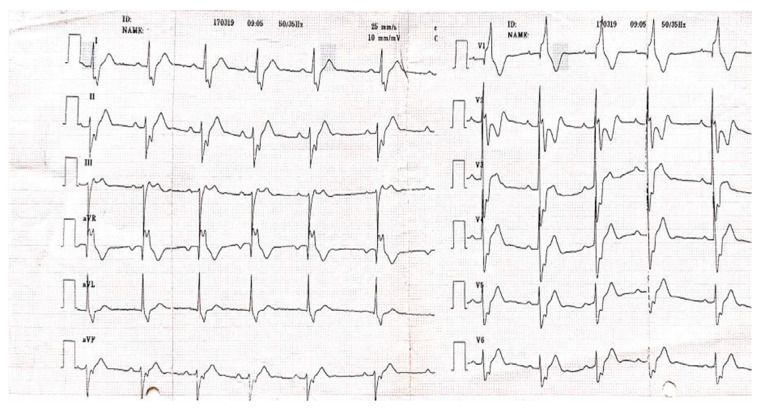
12-lead ECG with HR = 70 bpm, trifascicular block (first-degree AV block, left anterior fascicular block, right bundle branch block), PR interval = 240 ms, HR = 70 bpm Abbreviations: ECG—electrocardiogram, HR—heart rate; AV—atrioventricular.

A 24-year-old, non-smoker male patient, known with Ebstein’s anomaly and a trifascicular block (first-degree AV block, left anterior fascicular block, right bundle branch block) presented with dyspnea and mild effort intolerance. A physical examination revealed good general condition, BMI = 24.70 kg/m^2^, BP = 120/75 mmHg, HR = 75 bpm, and unremarkable cardiac and pulmonary auscultation. A 12-lead ECG revealed a trifascicular block (first-degree AV block, left anterior fascicular block, right bundle branch block) with a HR of 70 bpm (Figure 7).

**Figure 8:**
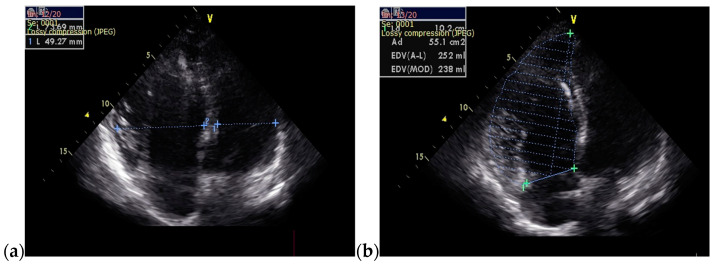

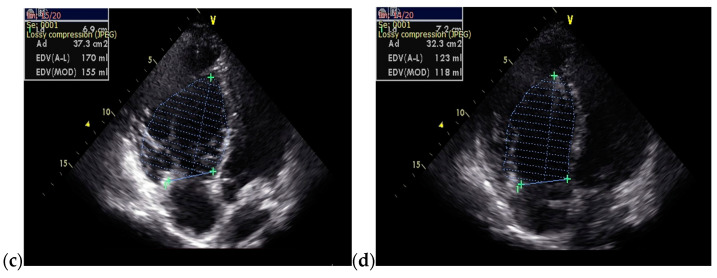
TTE on admission: (**a**) Apical 4-chamber view: dilated right cavities with a RV diameter of 7, 3 cm; (**b**) Apical 4-chamber view: total volume of the RV—252 mL; (**c**) Apical 4-chamber view: functional RV volume—170 mL; (**d**) Apical 4-chamber view: atrialized RV volume—123 mL; Abbreviations: TTE—transthoracic echocardiography; RV—right ventricle.

On admission, a TTE was performed and confirmed Ebstein’s anomaly. The main findings of the TTE were non-dilated left ventricle with an ejection fraction of 50% evaluated using Simpson’s method, dilated right chambers, atrialization of the RV, apical insertion of the septal cusp of the tricuspid valve, and mild functional tricuspid regurgitation (Figure 8).

**Figure 9:**
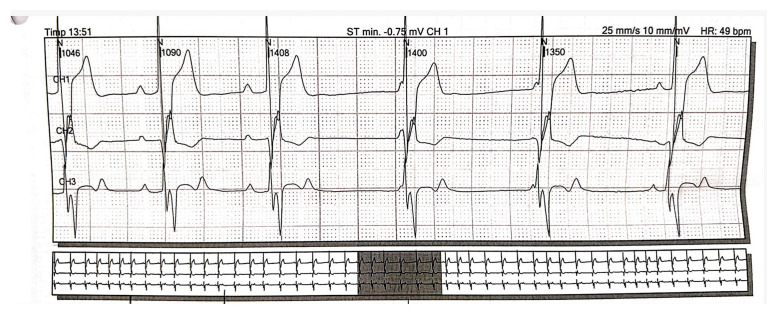
24-h Holter monitoring revealing paroxysmal third-degree AV block, HR = 49 bpm. Abbreviations: AV-atrioventricular; HR-heart rate.

During hospitalization, a 24-h ECG Holter monitoring was assessed and revealed a paroxysmal third-degree AV block (Figure 9).

According to current ESC Guidelines on cardiac pacing and cardiac resynchronization therapy, the patient was eligible for the cardiac pacemaker implantation (class of recommendation I, level of evidence C) due to a potential progression to permanent third-degree AV block and a high risk of syncope and sudden cardiac death.

**Figure 10:**
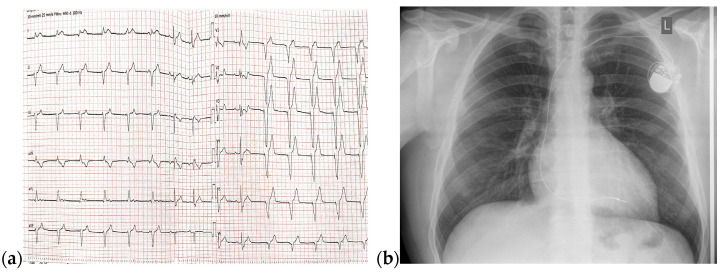
(**a**) 12-lead postprocedural ECG showed intermittent efficient ventricular paced rhythm; (**b**) Postero-anterior chest view radiography highlighting the presence of a single-chamber pacemaker and increased vascular density in the upper half of the hilum. Abbreviations: ECG—electrocardiogram.

Under local anesthesia, the implantation of a single-chamber VVI mode cardiac pacemaker was carried out. The programmed HR was 55 bpm. The postprocedural ECG showed intermittent efficient ventricular paced rhythm, with HR = 57 bpm (Figure 10a). The chest radiography revealed a single-chamber pacemaker and increased vascular density in the upper half of the hilum (Figure 10b).

The patient was discharged with improved symptomatology under diuretic therapy with furosemide and spironolactone.

**Figure 11:**
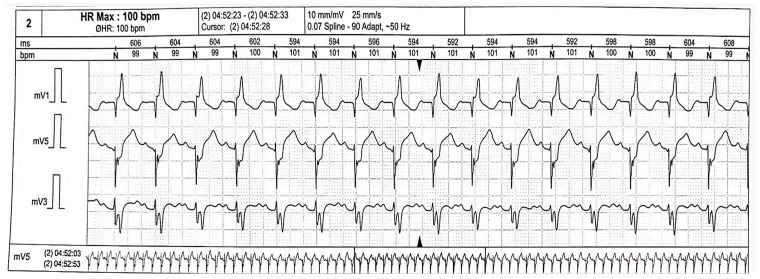
24-h Holter monitoring showing an episode of slow ventricular tachycardia during sleep.

Later on, the patient presented sudden-onset regular sustained rapid palpitations. A 24-h ECG Holter monitoring revealed multiple episodes of slow ventricular tachycardia with HR of 100 bpm (Figure 11). Therefore, antiarrhythmic treatment with propafenone was initiated.

**Figure 12:**
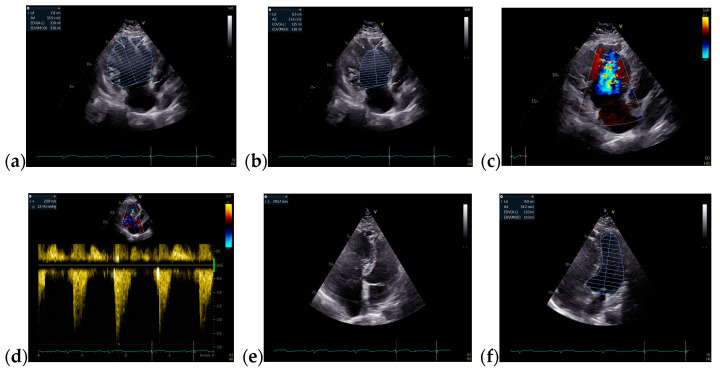
The TTE follow-up evaluation: (**a**) Apical 4-chamber view: dilated right cavities with a total volume of RV measuring 339 mL; (**b**) Apical 4-chamber view: atrialized RV volume of 135 mL; (**c**) Apical 4-chamber view: color Doppler flow revealing severe tricuspid regurgitation; (**d**) Tricuspid continuous wave Doppler flow: maximum RV-RA gradient = 33.45 mmHg; (**e**) Apical 4-chamber view: insertion of the septal cusp of the tricuspid valve at approximately 2.9 cm from the plane of the tricuspid annulus; (**f**) Apical 4-chamber view: end-diastolic left ventricular volume—110 mL. Abbreviations: TTE—transthoracic echocardiography; RV—right ventricle, RA—right atrium.

The follow-up TTE evaluation demonstrated a nondilated left ventricle with preserved ejection fraction, dilated right chambers, an increased total volume of RV measuring 339 mL and atrialized RV with a volume of 135 mL, severe functional tricuspid regurgitation, insertion of the septal cusp of the tricuspid valve approximately 2.9 cm from the plane of the tricuspid annulus and ventricular pacing electrode (Figure 12).

Since the pacemaker interrogation revealed multiple episodes of accelerated ventricular rhythm, the association with once-daily beta-blocker treatment to the antiarrhythmic therapy with propafenone was required. Furthermore, due to severe tricuspid regurgitation and mild PH, the furosemide dose was increased to 40 mg daily. With improved diuretic and antiarrhythmic treatment, the evolution of the patient was favorable without recurrence of symptoms.

## 4. Discussion

The first case presentation highlighted an isolated congenital pathology. In this case, the patient’s symptomatology occurred only when he reached adulthood. Similar situations were emphasized by studies that suggested that the diagnosis of atrial septal defects is increasingly common in adulthood, given the late onset of the symptoms, around the third decade of age. The patient’s symptomatology significantly improved after transcatheter device closure, indicating the importance of early diagnosis and treatment.

The second care presentation revealed the case of a patient with Ebstein’s anomaly who complained of worsening heart failure symptoms. A prompt evaluation of the patient through ECG Holter monitoring demonstrated a hidden rare pathology with a high risk of mortality. According to the literature data, the occurrence of third-degree AV block in association with Ebstein’s anomaly has rarely been reported, so a precise evaluation of CHD patients regarding the risk of late-onset complications is necessary.

Early diagnosis remains crucial for patients with CHD. During the last decades, due to constant improvement of diagnostic and therapeutic techniques, mortality rates among adult patients with CHD have decreased consistently. However, this category of patients has an increased risk of developing atrial tachyarrhythmias and, thus, thromboembolic risk requiring anticoagulant therapy. According to the recent data, for adult patients with CHD and atrial tachyarrhythmias, direct oral anticoagulants are equally effective and safe compared to vitamin K antagonists [50].

Clinical examination, ECG, and imaging remain essential diagnostic tools for patients with CHD, but another important issue is represented by the early identification of patients at risk of poor clinical outcomes. The previous research findings demonstrated that N-terminal-pro brain natriuretic peptide levels, lower prothrombin activity (<70%), and albumin levels (<40 g/L) indicate a high-risk status for poor clinical outcomes in patients with CHD [51].

## 5. Conclusions

Nowadays, the survival rate of patients with CHD has increased due to advanced treatment options and improved surgical techniques. The prognosis of these patients depends on the stage of the disease at the time of diagnosis, as well as on the association of symptoms and possible complications. Even if an increased number of CHD patients reach adulthood, the challenges related to early diagnosis and management, such as limited access to healthcare, low awareness of symptoms, and limited resources for diagnostic imaging tools, remain to be overcome.
